# Rapid testing for respiratory syncytial virus in a resource-limited paediatric intensive care setting

**DOI:** 10.4102/ajlm.v9i1.1084

**Published:** 2020-12-08

**Authors:** Howard Newman, Donald Tshabalala, Sikhumbuzo Mabunda, Nokwazi Nkosi, Candice Carelson

**Affiliations:** 1 National Health Laboratory Service, Virology, Port Elizabeth, South Africa; 2 Department of Pathology, Division of Medical Virology, Stellenbosch University, Cape Town, South Africa; 3 Faculty of Health Sciences, Nelson Mandela University, Port Elizabeth, South Africa; 4 Department of Paediatrics, Nelson Mandela Academic Hospital, Mthatha, South Africa; 5 Department of Paediatrics, Walter Sisulu University, Mthatha, South Africa; 6 Department of Public Health, Walter Sisulu University, Mthatha, South Africa; 7 Mpumalanga Department of Health, Nelspruit, South Africa; 8 National Health Laboratory Service, Tygerberg Academic Hospital, Cape Town, South Africa

**Keywords:** respiratory syncytial virus, rapid antigen tests, respiratory viruses, respiratory multiplex polymerase chain reaction, assay performance characteristics

## Abstract

We analysed the performance characteristics of the respiratory syncytial virus lateral flow rapid antigen assay in use when compared to a multiplex polymerase chain reaction for detection of respiratory viruses. The study was conducted at a tertiary paediatric hospital in Port Elizabeth, South Africa, from 01 January 2017 to 31 December 2018. We found the clinical sensitivity (36.8%) of the rapid test to be too low for routine diagnostic use. Knowledge of assay performance characteristics of rapid tests are important for appropriate interpretation of rapid test results.

## Introduction

Respiratory syncytial virus (RSV) infection is a common cause of casualty visits and hospital admissions in infants.^1,2,3,4^ In 2005, a meta-analysis reported that between 66 000 and 160 000 children under age 5 years died of RSV infection worldwide.^4^

During the South African RSV season, 50% – 60% of all respiratory admissions in children are due to RSV.^5^ Data from the 2016 pneumonia surveillance programme in South Africa showed that 17% of enrolled patients tested positive for RSV, with a case fatality rate of less than 1%.^6^

RSV is highly contagious and numerous hospital outbreaks have been reported in multiple age groups,^7^ thus necessitating appropriate infection prevention and control measures. For timeous initiation of appropriate infection prevention and control measures, rapid laboratory confirmation of RSV is essential.

Options for laboratory testing include virus isolation, rapid antigen tests, direct fluorescent antibody tests and molecular methods such as polymerase chain reaction (PCR).^8,9^ Rapid assays utilising antigen capture technology, that can be performed in less than 30 min, are available.^10^ Numerous commercial assays exist, ranging in sensitivity from approximately 50% to 96% in children, while the majority of assays have specificity above 97%.^1,11,12,13,14,15,16^ The sensitivity of rapid antigen assays for RSV is affected by several factors; decreased sensitivity was noted in patients with a prolonged duration of symptoms at the time of testing, infection with subtype B, and older age.^13,14,15,16^

Nucleic acid amplification tests have been shown to be superior to classical methods such as virus isolation and direct fluorescent antibody testing.^17,18^ Not only is PCR more sensitive and specific than conventional methods, but it is also more amenable to ‘multiplexing’, thus allowing for the simultaneous detection of a panel of common respiratory viruses.^19^ One limitation of molecular testing is its potentially prohibitive cost. This is of particular concern in resource-limited settings. In addition, the laboratory turn-around time may not be rapid enough to aid clinical decision making, especially when samples have to be referred to distant laboratories.^20^

The commercial rapid antigen RSV assay used in the virology laboratory of the National Health Laboratory Service in Port Elizabeth, South Africa, is the RSV Respi-Strip by Coris BioConcept (Gembloux, Belgium). It has a reported sensitivity of approximately 80%, with a specificity of greater than 95%.^16^ When compared to direct fluorescent antibody tests, the RSV Respi-Strip was found to be 92% sensitive and 98% specific.^21^ If the reported sensitivity of the assay of 80% is accurate, while not ideal, this may still be a clinically useful test in resource-limited settings, since positive cases would be detected 80% of the time, allowing for patient cohorting and isolation, and thereby limiting nosocomial transmission.^7^

For respiratory multiplex PCR testing, the virology laboratory in Port Elizabeth refers specimens to another National Health Laboratory Service virology laboratory at Tygerberg Hospital in Cape Town, South Africa. This laboratory makes use of the Seeplex^®^ RV16 assay by Seegene (Seoul, South Korea). The sensitivity and specificity for the individual viruses varies. For RSV, the sensitivity and specificity is reported to be above 90% when compared with singleplex or duplex PCR.^17^

Since patients admitted to our paediatric intensive care unit (ICU) for suspected lower respiratory tract infection are all tested for RSV with a rapid antigen assay, in addition to testing for a panel of common respiratory viruses (including RSV subtypes A and B) by PCR, we compared the two assays to determine the sensitivity and specificity of the RSV Respi-Strip assay. We additionally sought to describe the common respiratory viruses detected in our ICU setting.

## Methods

### Ethical considerations

This study received ethical clearance from the Human Research Committee of the Faculty of Health Sciences, Walter Sisulu University (reference 043/2018).

### Study design

This was a descriptive, retrospective cross-sectional study analysing laboratory reports for RSV rapid antigen and multiplex PCR tests for respiratory viruses. Results from the RSV rapid antigen assay were compared to results from the multiplex PCR assay, thus allowing for the calculation of performance characteristics of the rapid antigen assay. Factors associated with false-negative RSV rapid antigen results were analysed.

### Sample selection

The study population comprised all paediatric patients admitted to ICU at Dora Nginza Hospital in Port Elizabeth, Eastern Cape Province, South Africa, who had respiratory samples taken for laboratory investigation of viral infections from 1 January 2017 to 31 December 2018. Rapid testing for RSV is performed locally by the virology laboratory of the National Health Laboratory Service in Port Elizabeth, South Africa, with the nasopharyngeal aspirates being the specimen matrix. In addition, remnant specimen is referred to another virology laboratory (Cape Town, South Africa) within the National Health Laboratory Service, for multiplex PCR. This allowed for retrospective analysis of laboratory data.

### Data analysis

All variables were captured and coded in Microsoft Excel 2013 (Microsoft Corporation, Seattle, Washington, United States) and exported to Stata 14.1 for analysis (Stata Corp LP, College Station, Texas, United States). The distribution of age in days (numerical variable) was explored using the Shapiro Wilk test, and age was further converted into a categorical variable in months.

Clinical sensitivity and specificity of the RSV Respi-Strip rapid antigen assay was calculated by comparison against the multiplex PCR (Seeplex® RV16).

Categorical variables are presented using frequency tables, percentages and graphs. Bivariate logistic regression models were used to determine associations of false RSV rapid antigen results with risk factors such as age, sex, HIV co-infection, RSV subtype, and co-infections with other viruses or bacteria. The odds ratio was the relative measure of association used. The 95% confidence interval was used to estimate the precision of estimates. The level of statistical significance was set at 5% (*p*-value ≤ 0.05).

## Results

Test reports from a total of 152 participants were obtained and included in the study. Eighty-one of the participants (53.3%) were female, and 121 were under the age of one year. Table 1 shows all the demographic characteristics considered during this study.

**TABLE 1:** Demographic characteristics of the study population from Dora Nginza Hospital, Port Elizabeth, South Africa, 2017–2018.

Characteristic	*N*	Percentage	95% confidence interval
**Sex**			
Female	81	53.3	45.3–61.2
Male	71	46.7	38.8–54.7
**Age (months)**			
< 1	37	24.3	18.1–31.9
1–2	21	13.8	9.1–20.3
3–6	41	27.0	20.5–34.7
7–12	22	14.5	9.7–21.1
13–24	16	10.5	6.5–16.6
> 24	15	9.9	6.0–15.8
**HIV status**			
Positive	8	5.3	2.6–10.2
Negative	78	51.3	43.3–59.3
Not tested	66	43.4	35.7–51.5
**CMV respiratory PCR status**			
Positive	74	48.7	40.7–56.7
Negative	64	42.1	5.5–15.0
Not tested	14	9.2	34.4–50.2
**Viral (RSV)–bacterial co-infection**			
Yes	3	2.0	0.6–6.0
No	104	68.4	60.5–75.4
Not tested	45	29.6	22.8–37.4
**Viral–viral co-infection (RSV + any other virus)**			
Yes	13	8.6	5.0–14.3
No	139	91.4	85.7–95.0

*N* = 152.

CMV, cytomegalovirus; HIV, human immunodeficiency virus; PCR, polymerase chain reaction; RSV, respiratory syncytial virus.

**FIGURE 1:**
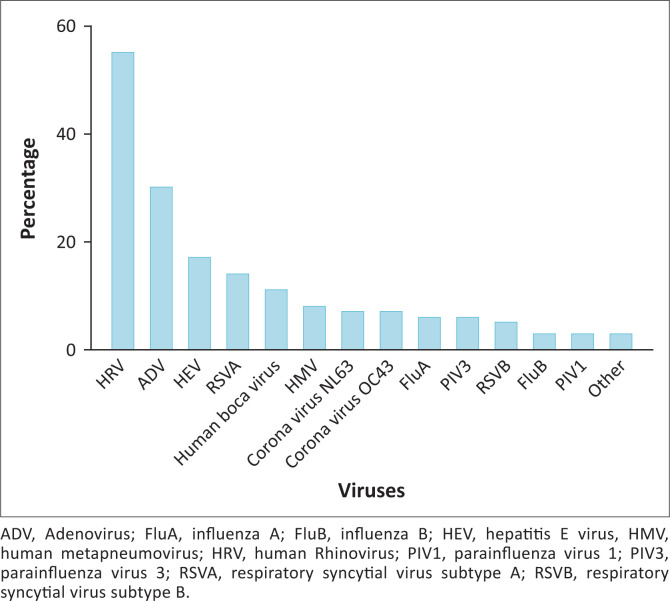
Prevalence of the various respiratory viruses detected in paediatric patients from Dora Nginza Hospital, Port Elizabeth, South Africa, 2017–2018.

Results from the multiplex PCR assay show that rhinovirus was the most common virus detected (*n* = 55), followed by adenovirus (*n* = 30), RSV (*n* = 19) and enterovirus (*n* = 17), with the remainder of the viruses occurring less commonly (Figure 1). Respiratory syncytial virus subtype A was detected much more frequently than subtype B.

When comparing the RSV rapid antigen assay to the multiplex PCR assay, the former was found to be 36.8% sensitive (Table 2).

**TABLE 2:** Retrospective analysis of laboratory data from Doran Nginza Hospital, Port Elizabeth, South Africa, 2017–2018.

Characteristic	Numerator/denominator	Percentage	Confidence interval
Sensitivity	7/19	36.8	16.3–61.6
Specificity	120/133	90.2	83.9–94.7
Positive predictive value	7/20	35.0	15.4–59.2
Negative predictive value	120/132	90.9	84.7–95.2

Note: Performance characteristics of the respiratory syncytial virus Respi-Strip by Coris BioConcept when compared to the multiplex polymerase chain reaction assay Seeplex^®^ RV16 by Seegene (which includes respiratory syncytial virus as a target).

PCR, polymerase chain reaction; RSV, respiratory syncytial virus.

When analysing factors associated with false-negative RSV rapid antigen results, only viral–viral co-infection and infection with RSV subtype A were statistically significant associations (Table 3).

**TABLE 3:** Factors associated with false-negative respiratory syncytial virus rapid antigen results from paediatric patients at Dora Nginza Hospital, Port Elizabeth, South Africa, 2017–2018.

Characteristic	OR	95% confidence interval	*p*
**Sex**			
Female	1.00	-	1.000
Male	1.29	2.45–8.14	0.563
**Age (months)**			
< 1	1.60	0.33–7.75	0.559
1–2	1.06	0.200–5.64	0.943
3–6	2.31	0.45–11.83	0.314
7–12	0.67	0.14–3.22	0.614
13–24	1.08	0.18–6.44	0.930
> 24	1.00	-	1.000
**CMV**			
Negative (on respiratory PCR)	1.00	-	1.000
Positive (on respiratory PCR)	1.74	0.65–4.66	0.274
**Viral (RSV)–bacterial co-infection**			
No	1.00	-	1.000
Yes	1.98	0.17–22.85	0.585
**Viral–viral co-infection (RSV + any other virus)**			
No	1.00	-	1.000
Yes	17.30	4.77–62.70	< 0.0001
**HIV**			
Negative	1.00	-	1.000
Positive	1.67	0.30–9.19	0.558
**RSVA**			
Negative	1.00	-	1.000
Positive	28.93	6.77–123.66	< 0.0001

OR, odds ratio; CMV, cytomegalovirus; HIV, human immunodeficiency virus; PCR, polymerase chain reaction; RSV, respiratory syncytial virus; RSVA, respiratory syncytial virus subtype A.

## Discussion

To the best of our knowledge, this is the first study comparing a RSV rapid antigen assay to a multiplex PCR assay that includes RSV as a target. In addition, it is also the first study to report on the prevalence of the common respiratory viruses found in our local paediatric ICU. We found the clinical sensitivity of the RSV rapid antigen assay (RSV Respi-Strip by Coris BioConcept) to be 36.84%; rhinovirus was the most commonly detected virus on the respiratory multiplex PCR assay. Although the majority of respiratory admissions worldwide are due to RSV,^5^ the most common viruses detected in our setting were rhinovirus (*n* = 55) and adenovirus (*n* = 30), followed by RSV (*n* = 19) and enterovirus (*n* = 17).

The main aim of this study was to determine the performance characteristics of the RSV rapid antigen assay in use (RSV Respi-Strip) by comparison to the respiratory multiplex PCR assay (Seeplex^®^ RV16 assay), which includes RSV as a target. The sensitivity of the RSV rapid antigen assay was found to be 36.84% (confidence interval: 16.29% – 61.64%). Even at the upper end of the confidence interval, this sensitivity is too low for routine diagnostic use. While the specificity of above 90% may be sufficient for a rapid antigen assay, the low sensitivity would have resulted in many cases being undiagnosed, thus defeating the main purpose of using a rapid test, which is to allow for early institution of infection prevention and control measures to mitigate against the well-known nosocomial outbreak risk.^7^ Potential reasons for the discrepancy in performance of this rapid test in our setting compared to the manufacturer’s claims are manifold. Rapid antigen assays are generally less sensitive than PCR. This can be compounded by low viral loads in the clinical samples, as may be the case with upper respiratory tract infections only. It was beyond the scope of this study to differentiate patients based on severity of disease. The relatively small sample size may also account for the discrepancy in clinical sensitivity.

The bivariate logistic regression analysis revealed a statistically significant association between false-negative RSV rapid antigen tests and viral co-infection, and infection with RSV subtype A. In contrast, a previous study showed that infection with RSV subtype B was associated with decreased sensitivity of rapid antigen assays.^15^

Cytomegalovirus and HIV positivity were more likely to be associated with false RSV rapid antigen results, as well as more likely to be associated with false-negative results specifically. Neither of these associations was statistically significant.

### Limitations

The main limitation of this study is the small sample size as a result of resource constraints, resulting in wider-than-ideal confidence intervals in the calculation of clinical sensitivity of the RSV rapid antigen test. However, even at the upper limit of the confidence intervals, the main conclusion that the sensitivity and positive predictive value are not acceptable for routine diagnostic use, still holds.

### Conclusion

This study highlights the importance of ongoing monitoring of newly introduced assays, as verification experiments may not always determine whether an assay will perform optimally in real-world conditions. Based on the results of this study, the RSV rapid antigen assay in use (RSV Respi-Strip) was discontinued due to an unacceptably high rate of false results.

In conclusion, we have demonstrated the common respiratory viruses found in our local paediatric ICU setting. In addition, we report on the poor performance of the RSV rapid antigen assay in use and the potential factors associated with false results, with only viral co-infections and infection with RSV subtype A being statistically significant.

Clinicians should have an idea of the sensitivity and specificity of the rapid tests in use to allow for the appropriate interpretation of results.
